# Age-Related Dysphagia Among Children with 22q11.2-Deletion Syndrome

**DOI:** 10.1177/10556656251345211

**Published:** 2025-05-28

**Authors:** Taylor B. Teplitzky, Neila L. Kline, Ashley F. Brown, Kandi Trevino, Caitlin Wilson, Cortney Van’t Slot, Yann-Fuu Kou, Romaine F. Johnson, Stephen R. Chorney

**Affiliations:** 1 Department of Otolaryngology, Nemours Children's Hospital, Wilmington, Delaware, USA; 2 Department of Otolaryngology-Head and Neck Surgery, University of Texas Southwestern Medical Center, Dallas, Texas, USA; 3 Department of Pediatric Otolaryngology, Children's Medical Center Dallas, Dallas, Texas, USA

**Keywords:** pediatric dysphagia, 22q11.2-deletion syndrome, DiGeorge syndrome, pediatric videofluoroscopic swallow study

## Abstract

**Objective:**

To describe videofluoroscopic swallow study (VFSS) findings for children with 22q11.2-deletion syndrome and evaluate age-associated changes.

**Design:**

Retrospective case series.

**Setting:**

Tertiary children's hospital.

**Patients, Participants:**

Children <18 years old.

**Methods:**

All VFSS between 2016 and 2021 were included. Comorbidities and dysphagia patterns were compared between children younger or older than 2 years of age.

**Interventions:**

None.

**Main Outcome Measures:**

Abnormal swallowing findings on VFSS measured by a standardized severity scale of oral and pharyngeal dysphagia.

**Results:**

40 children obtained a VFSS at a mean age of 4.3 years (standard deviation (SD): 5.5). Congenital heart disease was seen in 78% (N = 31) and gastroesophageal reflux disease in 50% (N = 20). Oropharyngeal dysphagia was demonstrated in 98% (N = 39). Oral residue was seen in 30% (N = 12) and 28% (N = 11) had pharyngonasal reflux. Pharyngeal residue was noted in 75% (N = 30), abnormal pharyngeal squeeze in 55% (N = 22), abnormal hyolaryngeal elevation in 45% (N = 18), delayed swallow in 45% (N = 18), and pharyngeal pooling in 15% (N = 6). Thin-liquid aspiration was identified in 35% (N = 14). A modified diet was required for 75% (N = 30) and 38% of children (N = 15) also required tube feeding. When comparing the children less than 2 years (53%, N = 21), pharyngeal squeeze abnormalities were less common compared to older children (33% vs. 79%, *P* = .01).

**Conclusion:**

Oropharyngeal dysphagia is common in children with 22q11.2-deletion syndrome characterized by abnormal pharyngeal squeeze and hyolaryngeal elevation with delayed swallow triggers. While most maintained modified oral intake, age-related changes were not encountered on VFSS.

## Introduction

22q11.2-deletion syndrome, also called velo-cardio-facial or DiGeorge syndrome, is the most common chromosomal deletion syndrome affecting 1 in 2000-7000 births.^1,2^ The phenotype of 22q11.2-deletion syndrome is variable, with over 180 physical and behavioral features reported.^2^ For this reason, presentation is inconsistent and diagnosis is confirmed via genetic testing. Some of the more common findings include dysmorphic craniofacial features, cardiac anomalies and conotruncal defects, immunodeficiencies from thymic hypoplasia, parathyroid dysfunction and hypocalcemia, velopharyngeal insufficiency, cleft palate, developmental delay, esophageal dysmotility, behavioral and psychiatric diagnoses, and swallowing disorders.^1,3^

Pediatric dysphagia can be related to functional or anatomic challenges and is characterized by difficult or abnormal swallowing of liquids, solids, or both. This can lead to aspiration, the inhalation of material into the lower airway, which may result in significant respiratory morbidity.^4^ In infants, dysphagia may be related to poor coordination, neurologic impairments and hypotonia, or anatomic abnormalities affecting the oral cavity and oropharynx.^1,5^ Animal models of 22q11.2-deletion syndrome have demonstrated central abnormalities including altered hindbrain patterning, neural crest cell migration, and changes to cranial nerve growth leading to altered development of the neural circuits needed for swallowing.^6^ Despite the well-established incidence of dysphagia in children with 22q11.2-deletion syndrome,^1,5,7,8^ the pattern and progression of swallow dysfunction remains ill-defined. The goal of this study is to describe videofluoroscopic swallow study (VFSS) findings for children with 22q11.2-deletion syndrome, and to evaluate for age-associated changes.

## Methods

A case series with chart review of all VFSS studies in children with 22q11.2-deletion syndrome at Children's Medical Center Dallas was approved by the University of Texas Southwestern Medical Center Institutional Review Board (STU-2018-0284). All VFSS between January 1, 2016 and December 31, 2021 were included. Demographics, comorbidities, and patterns of dysphagia were evaluated.

The VFSSs were performed by speech language pathologists (SLPs) who specialize in pediatric care. At this facility, a pediatric radiology technician was present in the room, but a pediatric radiologist was available for consultation. There are approximately 8 trained SLPs and they record images at 30 frames per second. Afterwards, the images were reviewed typically with a second SLP to analyze the findings using playback. Children were provided with age-appropriate contrast materials. Consistencies included thin liquids, nectar thick liquids, purees, and solids. Choice of foods to test was determined by the SLP based on feeding experience, medical history, and age of the child. The following specific findings on VFSS were attributed to weak or poorly coordinated muscular movements: reduced tongue elevation or retraction, laryngeal elevation, hyolaryngeal excursion, and pharyngeal squeeze. Delayed trigger is typically reader dependent, and variable based on a particular SLP. However, our group defines this as a delay in swallow that impacts safety of swallow or ability to protect the airway. Residue was any residual contrast material (not well-defined in pediatrics).

A well-defined scale was used for varying severity of dysphagia which is standardized across SLPs reading a VFSS. While no validated evaluation forms are used by SLPs, there is a well-defined assessment scale that it is standardized (Supplemental Table 1). The severity rating scale was developed at our institution by subject matter experts.

To assess changes over time, VFSS characteristics were compared using Pearson χ^2^ test after grouping children by age over or under 2 years at the time of the study. This age was chosen to delineate our two groups as previous studies have demonstrated infantile dysphagia resolution in 76% of children by age 2 years.^9,10^

All statistics were performed with Stata (StataCorp. 2021. *Stata Statistical Software: Release 17*. College Station, TX: StataCorp LLC.). Continuous data are presented as means with standard deviations. Categorical data are presented as counts with percentages. Statistical analysis was performed using Chi squared testing for categorical data or student's t test for continuous data. Statistical significance was set at *P* < .05.

## Results

A total of 40 children were diagnosed with 22q11.2-deletion syndrome and underwent a VFSS during the study period. Table 1 shows the demographics and comorbidities of the included children. Cardiac anomalies were seen in N = 31 (78%), while comorbid gastroesophageal reflux disease (GERD) was noted in N = 20 (50%) and neurodevelopmental delay in N = 21 (52.5%). At the time of the study, N = 30 (75%) children were taking a modified oral diet, and N = 15 (38%) used a feeding tube (either nasogastric tube or gastrostomy tube). Oral phase VFSS findings are shown in Table 2. Almost all children N = 39 (98%) demonstrated oral phase dysphagia, with equal numbers across dysphagia severities. The most common oral phase findings were abnormal tone in the oral tongue (N = 19, 48%) and cheeks (N = 21, 53%).

**Table 1.** Demographics of Children with 22q11.2-Deletion Syndrome.

Variable	Value
Age, mean yr (SD)	4.3 (5.5)
Age, median yr (Interquartile range, IQR)	1.7 (0.5-5.3)
Age <2 years, n (%)	21 (53)
Gestational age, mean wk (SD)	36.7 (2.2)
Gestational age, median wk (IQR)	37 (36-38)
Birth weight, mean g (SD)	2726.4 (646.1)
Birth weight, median g (IQR)	2652.5 (2495-3260)
Short gestational age <37 wk, n (%)	17 (43)
Male sex, n (%)	23 (58)
Race, n (%)	
White/Caucasian	25 (63)
Black/African American	11 (28)
Other	4 (10)
Hispanic ethnicity, n (%)	14 (35)
Comorbidities, n (%)	
Gastroesophageal reflux disease	20 (50)
Vocal cord paralysis	7 (18)
Cardiac anomaly	31 (78)
Neurodevelopmental diagnosis	21 (53)
Modified diet, n (%)	30 (75)
Use of feeding tube, n (%)	15 (38)

SD, standard deviation.

**Table 2.** Oral Phase VFSS Findings in Children with 22q11.2-Deletion Syndrome.

VFSS variable	Value, n (%)
Oral phase dysphagia	39 (98)
Oral phase dysphagia severity	
Functional	10 (26)
Mild	10 (26)
Moderate	11 (28)
Severe	8 (21)
Nasal reflux	11 (28)
Abnormal labial tone	15 (38)
Abnormal oral tongue tone	19 (48)
Abnormal cheek tone	21 (53)
Submucosal cleft palate	2 (5)
Oral residue present	12 (30)

VFSS, videofluoroscopic swallow study.

Table 3 reports the pharyngeal phase VFSS findings. Almost all (N = 39, 98%) were found to have pharyngeal phase dysphagia, with more children showing mild (N = 12, 30%) and moderate (N = 13, 32%) severity, versus functional (N = 6, 15%) or severe (N = 9, 23%) dysphagia. Aspiration was uncommon (N = 14, 35%), as was pharyngeal pooling (N = 6, 15%). Almost half of children with a delayed swallow trigger (N = 18, 45%), and an associated abnormal hyolaryngeal elevation was found in N = 18 (45%). Pharyngeal squeeze was abnormal in 22 patients (55%), pharyngeal residue in 30 (75%), and abnormal base of tongue mobility in 22 children (55%). VFSS findings were then compared by age < 2 years or ≥ 2 years, with results demonstrated in Table 4. There were equal numbers of children in each age group without a significant association between the VFSS features and age.

**Table 3.** Pharyngeal Phase VFSS Findings in Children with 22q11.2-Deletion Syndrome.

VFSS variable	Value, n (%)
Pharyngeal phase dysphagia	39 (98)
Pharyngeal phase dysphagia severity	
Functional	6 (15)
Mild	12 (30)
Moderate	13 (32)
Severe	9 (23)
Aspiration present	14 (35)
Pharyngeal pooling	6 (15)
Abnormal hyolaryngeal elevation	18 (45)
Delayed swallow trigger	18 (45)
Abnormal pharyngeal squeeze	22 (55)
Pharyngeal residue	30 (75)
Abnormal base of tongue mobility	22 (55)

VFSS, videofluoroscopic swallow study.

**Table 4.** Findings on VFSS in Children with 22q11.2-Deletion Syndrome Based on Age.

Variable	Age < 2 years (n = 21)	Age ≥ 2 years (n = 19)	*P-*value
Oral phase dysphagia, n (%)	20 (93)	19 (100)	.34
Oral residue present, n (%)	14 (67)	14 (74)	.63
Pharyngeal phase dysphagia, n (%)	20 (95)	19 (100)	.34
Pharyngeal residue present, n (%)	14 (70)	16 (84)	.20
Base of tongue abnormality, n (%)	10 (50)	12 (63)	.38
Nasal reflux, n (%)	7 (35)	4 (21)	.39
Aspiration, n (%)	9 (45)	5 (24)	.27
Modified diet, n (%)	16 (80)	14 (74)	.86
Tube feeding, n (%)	9 (45)	6 (32)	.46

VFSS, videofluoroscopic swallow study.

## Discussion

This study evaluated specific VFSS characteristics in children with 22q11.2-deletion syndrome, as well as differences in swallowing features with respect to age. Dysphagia is a recognized feature of 22q11.2-deletion syndrome occurring in 30-40% of children.^5,7,8^ Previous studies have characterized the patterns of dysphagia in this population during infancy,^1^ as well as the presence of dysphagia as a prominent otolaryngologic manifestation.^8^ The present study focused on children with subjective swallow dysfunction who were referred for formal testing. In this cohort, dysphagia was characterized by abnormal pharyngeal squeeze and hyolaryngeal elevation with delayed swallow triggers though a majority were able to sustain an oral diet.

The etiology of dysphagia in children with 22q11.2-deletion syndrome is poorly understood. Feeding difficulty may arise from uncoordinated tongue, pharyngeal, and esophageal musculature that can be centrally or peripherally neurologic in origin.^10,11^ Additionally, cardiac defects leading to dyspnea can impact feeding.^10^ It has been well established that the presence of a cardiovascular anomaly and congenital heart disease is associated with pediatric dysphagia.^12,13^ In our cohort, a majority had a cardiac anomaly, corroborating the association in this population. Additionally, half of children had GERD, which also is well described in this patient population.^5,8,14^ GERD is a common finding in children with dysphagia,^15,16^ with swallow dysfunction playing a role in clearance of reflux episodes.^16^ In these children, GERD may also be related to global hypotonia.^10^ The esophageal phase of swallowing was not universally evaluated on the VFSS. Additionally, pH probe testing information was not gathered. This poses an area of future research to help delineate the role, and cause, of GERD in this population, and its implications on swallow dysfunction.

Oral phase dysphagia was present in all but one child. The most common issues were potentially related to hypotonia, with poor oral tongue and cheek tone, seen in about half of children. This finding contrasts previous reports, with a normal oral phase of swallowing in this population.^5^ In addition, children with 22q11.2-deletion syndrome have difficulty with neuromuscular control.^17,18^ Therefore, it is fitting that a key feature of the oral dysphagia is weakness and poor coordination. Interestingly, only two children were noted to have a submucosal cleft palate and a minority had nasal reflux. This contrasts with the literature where nasopharyngeal reflux is frequent, ranging from 50 to 75% of children with 22q11.2-deletion syndrome.^1,5,19,20^ Additionally, nasal reflux is common in the setting of submucosal cleft palate.^21^ The reason for the lower rate of submucosal cleft palate in our population is unclear, but may speak to the vast range of phenotypic presentations seen in 22q11.2-deletion syndrome. Implications on speech, namely hypernasal speech, and recurrent ear infections from eustachian tube dysfunction are also common among children with submucosal cleft palates.^21^ Our study did not evaluate these additional features in the population with dysphagia. It is possible that children with velopharyngeal insufficiency in the setting of a submucosal cleft palate have a different complement of findings than those with clinically significant dysphagia. The incidence of speech and ear disorders in these children with objective assessments of dysphagia is an area of continued research.

The pharyngeal phase of swallowing was commonly impacted. Almost all children had an abnormal pharyngeal phase swallow on the VFSS with half having abnormal hyolaryngeal elevation. Additionally, over half were noted to have abnormal pharyngeal squeeze and decreased mobility of the base of tongue. An important irregularity was a high incidence of delayed swallow trigger, with a majority having persistent pharyngeal residue, suggesting altered sensation. Abnormal sensation plays a recognized role in swallowing dysfunction in children. For children with 22q11.2-deletion syndrome, there are recognized reductions in sensory thresholds, and these are likely associated with more severe swallowing measures including delayed swallow. Children with dysphagia are known to have impaired laryngopharyngeal sensation.^22^ It has also been described how GERD can alter laryngeal sensitivity,^23,24^ and GERD was prevalent in our study, which is recognized in children with 22q11.2-deletion syndrome.^14,25^ Importantly, aspiration was rare in our cohort, which is in contrast to previous reports noting 55% of children with 22q11.2-deletion with evidence of aspiration.^20^ This is interesting, given the aforementioned issues around sensation. However, low rates of aspiration fits with an ability to maintain an oral diet, albeit a modified diet as seen in the majority of our patients, as thickening of feeds can improve dysphagia in the setting of GERD.^23^ A fair number of children in our cohort did use a feeding tube. Rates of gastrostomy tube use in this population varies, with some articles citing low incidences around 8-16%,^25^ with others noting 50-60%.^5,20^ Our cohort adds to the existing literature regarding gastrostomy tube rates.

An important finding of our study was that as children aged, their dysphagia persisted. There were no significant differences in the studied VFSS features when children were compared either over or under age 2 years. Kotcher et al^25^ found dysphagia remains a prevalent issue in children less than age 13 and is noted in approximately 39%, though with improvement from childhood to adolescence. The persistence of neuromuscular deficits in this population^17,18^ may contribute to the persistence of symptoms. However, it is possible that longer follow-up periods in our cohort may demonstrate improvement or resolution of dysphagia over time.

There are several limitations of this study which are important to discuss. First, this cohort derives from a single institution which may limit the generalizability of our findings. Secondly, there are characteristic flaws in a retrospective study with inherent selection bias. Third, though 40 children are a sizeable group with this genetic condition, it is a small sample size. A possible way to improve upon this would be to consider a multi-institutional assessment of children with 22q11.2-deletion syndrome for a more robust analysis. While only thin-liquid aspiration was noted, there was no analysis of non-thin consistency dysphagia since the decisions to thicken can be varied based on patient factors as well as SLP practices. Fourth, an inherent limitation in analyzing pediatric VFSS is the potential for interpretation biases due to variation in assessment scales utilized. Development of a standardized analysis tool would be an important area of future research to help improve the robustness of research in this field. Finally, while it is unclear what the prevalence of dysphagia is among all children with 22q11.2-deletion syndrome, it is presumed that most, but not all, have some component of dysphagia. This limitation warrants further studies where more routine VFSS are obtained on this population of children.

Overall, this study adds important insights into the characteristics of dysphagia in children with 22q11.2-deletion syndrome. Abnormalities related to the oral and pharyngeal phases of swallowing are pervasive, with evidence of both abnormal pharyngeal muscular control and decreased sensation. Dysphagia remains stable over time, without significant differences when children were compared by age.

## Conclusion

Oropharyngeal dysphagia is common in children with 22q11.2-deletion syndrome. Formal evaluation of swallowing via VFSS demonstrates oral and pharyngeal phase abnormalities characterized by weak oropharyngeal muscular movements with discoordination and decreased pharyngolaryngeal sensation. Aspiration and gastrostomy were both identified among this group of children with a majority maintaining an oral diet via modifications. Patterns of dysphagia are persistent, without significant age-related changes on VFSS testing.

## Supplemental Material

sj-docx-1-cpc-10.1177_10556656251345211 - Supplemental material for Age-Related Dysphagia Among Children with 22q11.2-Deletion Syndrome Supplemental material, sj-docx-1-cpc-10.1177_10556656251345211 for Age-Related Dysphagia Among Children with 22q11.2-Deletion Syndrome by Taylor B. Teplitzky, Neila L. Kline, Ashley F. Brown, Kandi Trevino, Caitlin Wilson, Cortney Van’t Slot, Yann-Fuu Kou, Romaine F. Johnson and Stephen R. Chorney in The Cleft Palate Craniofacial Journal
