# Supplementary material for: Age-Related Dysphagia Among Children with 22q11.2-Deletion Syndrome
Source: Cleft Palate Craniofac J. 2025 May 28;63(6):1661–6. doi: 10.1177/10556656251345211 (PMC13176480; doi:10.1177/10556656251345211)
Supplement: sj-docx-1-cpc-10.1177_10556656251345211 - Supplemental material for Age-Related Dysphagia Among Children with 22q11.2-Deletion Syndrome [file sj-docx-1-cpc-10.1177_10556656251345211.docx]

**Supplemental Table 1:** Severity Rating Scales

| Oral Phase Severity Scales | |
| --- | --- |
| Functional | Skills are normal for meeting nutritional needs with an appropriate diet in all situations. No dietary modifications or compensatory strategies required. |
| Mild | Patient able to take all of nutrition by mouth. General feeding strategies needed only. Adequate intake without intervention. No modification in consistencies recommended. Patient’s intake adequate for appropriate weight gain. |
| Moderate | Patient able to take all of nutrition by mouth. Specific feeding strategies recommended. Intake requires intervention. Decreased intake may be impacting weight gain. Modifications in consistencies may be recommended. |
| Severe | Patient is unable to take all nutrition by mouth requiring alternative means of nutrition. Alternative means of nutrition is needed to supplement nutritional needs. Intake requires intervention. Decreased intake impacting weight gain. Modifications in consistencies may be recommended. |
| Profound | Patient takes all of nutrition by alternative means of nutrition. Intake requires intervention. Minimal PO trials or non-nutritive oral motor stimulation recommended on a trial basis by a trained caregiver. Patient may not tolerate own secretions. Complete NPO status may be recommended. |
| Pharyngeal Phase Severity Scales | |
| Functional | Swallowing is normal for meeting nutritional needs with an appropriate diet in all situations. No dietary modifications or compensatory strategies required. |
| Mild | Swallowing is normal for meeting nutritional needs with an appropriate diet in all situations but minimal compensatory techniques/safety precautions may be needed as well as additional time. |
| Moderate | Swallowing is adequate/functional for meeting nutritional needs with a simplified or modified diet, and supervision to ensure consistent use of compensatory techniques/safety precautions. Secretion management may be decreased. Autonomic responses may interfere with the feeding process and require supervision and/or intervention. |
| Severe | Swallowing is possible but not for nutritional needs. Secretion management is frequently diminished. Hypersensitivity with or without gag/cough/vomit responses frequently interfere with feeding. Autonomic responses are always present and limit oral feeding to a minimum. |
| Profound | Swallowing is not functional for nutrition or for secretion management. Protective reflexes may not be present or may be so strong as to preclude feeding. Severe autonomic responses may be noted. No oral feeding due to risk of aspiration. |
